# Group Cognitive-Behavior Therapy or Group Metacognitive Therapy for Obsessive-Compulsive Disorder? Benchmarking and Comparative Effectiveness in a Routine Clinical Service

**DOI:** 10.3389/fpsyg.2018.02551

**Published:** 2018-12-10

**Authors:** Costas Papageorgiou, Karen Carlile, Sue Thorgaard, Howard Waring, Justin Haslam, Louise Horne, Adrian Wells

**Affiliations:** ^1^The Priory Hospital Altrincham, Altrincham, United Kingdom; ^2^Mersey Care NHS Foundation Trust, Ashworth Hospital, Liverpool, United Kingdom; ^3^Greater Manchester Mental Health NHS Foundation Trust, University of Manchester, Manchester, United Kingdom

**Keywords:** cognitive-behavior therapy, metacognitive therapy, group therapy, obsessive-compulsive disorder, benchmarking, effectiveness, routine practice

## Abstract

Cognitive-behavior therapy (CBT), delivered in an individual or group format, is the recommended treatment of choice for Obsessive-Compulsive Disorder (OCD), but no studies have benchmarked the outcomes for group CBT in real-world clinical settings. The first aim of this evaluation was to benchmark the outcomes for group CBT in a sample of 125 patients who attended a routine clinical service for OCD. The results showed that the outcomes for the group CBT were comparable to those reported in previous treatment studies. However, consistent with the CBT for OCD literature, 28% of patients receiving CBT reported minimal improvement. The second aim of this evaluation was to carry out a benchmarking analysis for group metacognitive therapy (MCT) to determine if this could provide any advantages in a sample of 95 patients who also attended this clinical service over a subsequent period. The clinically significant results obtained for group MCT improved upon or equaled those obtained for group CBT and those typically found in treatment studies. The group MCT cohort improved significantly more than the group CBT cohort even after controlling for important pre-treatment variables including age, gender, number of diagnoses, symptoms of depression, and psychotropic medication. MCT had significantly higher clinical response rates. Based on international expert consensus criteria, 86.3% of patients in the MCT cohort responded compared with 64% in CBT. The implications of these findings are discussed.

## Introduction

Obsessive-Compulsive Disorder (OCD) is a common, debilitating, and chronic mental health problem. Epidemiological studies have estimated the lifetime prevalence of OCD to be approximately 2%, with most individuals with OCD being affected before their mid-twenties (Kessler et al., 2005). OCD has been ranked among the 10 most debilitating disorders in the world (World Health Organization, 1999). Once developed, OCD tends to have a continuous course in the majority of individuals (84%) and deteriorating (14%) or episodic (2%) courses in others (Rasmussen and Tsuang, 1986). Therefore, in the absence of effective treatment, OCD can persist for many years causing significant functional impairments and reduced quality of life (Koran et al., 1996).

The currently recommended psychological treatment of choice for OCD is cognitive-behavioral therapy (CBT; National Institute for Health Clinical Excellence, 2005), which comprises exposure and response prevention (ERP) with or without OCD-focused cognitive therapy (CT). Meta-analytic studies on the effects of psychological treatments for OCD have concluded that CBT has the highest degree of empirical support (e.g., Rosa-Alcázar et al., 2008; Olatunji et al., 2013; Öst et al., 2015). Öst et al. (2015) conducted the most recent and extensive meta-analysis, which included all randomized controlled trials (RCTs) of CBT for OCD, and the results supported the effectiveness of ERP with or without specific CT strategies with very large effect sizes (ES) for the comparisons of CBT with waiting list (1.31) and placebo conditions (1.33). In addition, other previous meta-analyses focusing only on group CBT for OCD found a mean ES of 1.12 compared with waiting list, which indicates that group CBT is an effective format (Jónsson and Hougaard, 2009). Of particular relevance to the present paper, the results of Öst et al. (2015) also showed that the ES for the comparisons between individual and group interventions (0.17) were small and non-significant. Therefore, the empirical evidence shows that CBT is currently the most effective psychological intervention for OCD and the format of CBT (i.e., individual or group) does not affect its outcome.

Whilst the efficacy of CBT for OCD has been established through a number of RCTs, which possess strong internal validity, the generalizability of the findings from these research studies to routine clinical practice is rather limited due to the rigid methodological features of such experimental designs. A central tenet of evidence-based healthcare is a requirement for the objective evaluation of health service interventions for their provision in clinical practice (Sackett et al., 1996). Given the phenomenological characteristics of OCD, such as chronicity and comorbidity (Kessler et al., 2005), it is imperative to determine whether the results from RCTs may be translated into real-world clinical settings. An effective method of achieving this is through benchmarking, which is a type of clinical audit that seeks to examine and improve the quality of treatment by comparing outcomes of a routinely delivered clinical service to those obtained in RCTs. To date, there have been only a few published studies that have benchmarked outcomes for CBT for adults with OCD (Franklin et al., 2000; Rothbaum and Shahar, 2000; Warren and Thomas, 2001; Houghton et al., 2010). Collectively, these studies provide some initial evidence of outcomes comparable to those falling within the benchmarks derived from previous relevant RCTs, but these few studies have a number of key limitations such as small sample sizes of self-selected participants and comparisons with only a limited number of RCTs. Importantly, considering the documented cost and clinical effectiveness of group CBT for OCD, none of the CBT interventions reported in the published benchmarking studies appear to have been delivered in group formats. Therefore, our first aim was to carry out a systematic benchmarking analysis of the treatment outcomes for group CBT for adults who had attended a routine clinical service for OCD in a mental health hospital over a 5-year period. Subsequently, in view of the results of this benchmarking analysis, our second aim was to examine the relative effectiveness associated with introducing an alternative psychological treatment approach: metacognitive therapy (MCT; Wells, 2009), which was also delivered in group formats, and to systematically benchmark this approach. The introduction and evaluation of alternative treatments is clearly supported by the literature that shows that more than a third of patients with OCD have a minimal or no response to CBT or continue to have significant residual symptoms (e.g., Wilhelm, 2000; Fisher and Wells, 2005b).

Metacognitive therapy for OCD developed from a specific metacognitive model of OCD (Wells, 1997, 2009), which was originally grounded on the generic Self-Regulatory Executive Function model (Wells and Matthews, 1994, 1996), where metacognition has prominence in explaining the development and maintenance of emotional disorders. According to the metacognitive model of OCD (Wells, 1997), the experience of intrusive thoughts, which are both universal phenomena but also cardinal clinical features of OCD, is linked with underlying metacognitive beliefs which in turn guide maladaptive thinking referred to as the cognitive attentional syndrome (CAS). The two domains of metacognitive beliefs include (1) beliefs about the significance or dangerousness of intrusive thoughts/feelings and (2) beliefs about the need to perform rituals. The first domain of metacognitive beliefs, also termed fusion beliefs, include: thought-event fusion, the belief that the occurrence of a thought can cause events to happen or that an event has already happened; thought-action fusion, the belief that thoughts alone can make a person carry out unwanted actions or behaviors; and thought-object fusion, the belief that thoughts or feelings can be transferred into objects. Metacognitive beliefs lead to worry and rumination in response to inner cognitive events (e.g., intrusive thoughts), resulting in sustained emotional distress. The second domain, beliefs about rituals, guide responses to these worries and can be expressed in a declarative form (e.g., “I must wash until I stop thinking about germs”) or as a plan for monitoring action, which is indicated by a stop criterion or a “stop signal.” In Wells’ metacognitive model of OCD, the CAS consists of worry, rumination, threat monitoring, and maladaptive behaviors in the form of overt and covert rituals, all of which serve as means of coping with worry linked to obsessions. Whilst this model may be considered as an appraisal theory of OCD, it is distinct in that the nature of the negative appraisal is defined by the CAS and beliefs are solely metacognitive. In contrast, in CBT multiple belief domains are involved including inflated responsibility (Salkovskis, 1985, 1999; Rachman, 1993), intolerance of uncertainty (Carr, 1974), perfectionism (Frost and Steketee, 1997), overestimation of threat and importance of and need to control thoughts (Obsessive Compulsive Cognitions Working Group, 1997). MCT does not prioritize these beliefs but focuses only on metacognitive beliefs about thoughts and beliefs about rituals. CBT does not formulate beliefs about rituals.

Cross-sectional, prospective, and experimental studies in both clinical and non-clinical populations provide support for the metacognitive model of OCD. Metacognitive beliefs in general, and fusion beliefs in particular, correlate positively with OCD symptoms in non-clinical samples (Cartwright-Hatton and Wells, 1997; Wells and Papageorgiou, 1998; Emmelkamp and Aardema, 1999; Sica et al., 2007). Furthermore, metacognitive beliefs are stronger predictors of OCD symptoms than cognitive beliefs, such as responsibility, intolerance of uncertainty, perfectionism, which explain little or no additional variance (Gwilliam et al., 2004; Myers and Wells, 2005; Myers et al., 2009a). In a prospective study, Myers et al. (2009b) found that, when statistically controlling for worry and overestimation of threat, only fusion beliefs emerged as a significant independent predictor of obsessive-compulsive symptoms, but other beliefs did not. In a routine treatment study, Solem et al. (2009) found that changes in metacognitive beliefs were a better predictor of outcomes than changes in responsibility and perfectionism among patients receiving ERP even after controlling for cognitive factors. Subsequently, Grøtte et al. (2015) replicated and extended this study by using a larger clinical sample and specific measures of metacognition assessing fusion beliefs and beliefs about rituals. Therefore, there is considerable empirical evidence to support the metacognitive model of OCD and the specific and direct role that metacognition plays over and above cognition.

Metacognitive therapy for OCD (Wells, 1997, 2009) directly focuses on modifying metacognitive beliefs and beliefs about rituals. Empirical evidence supporting MCT for OCD has derived from experimental component studies (Fisher and Wells, 2005a). Evidence supporting full MCT for OCD has derived from single case series in children and adolescents (Simons et al., 2006) as well as adults receiving this treatment in both an individual (Fisher and Wells, 2008; Van der Heiden et al., 2016) and group (Rees and van Koesveld, 2008) format. In addition, an RCT comparing individual MCT for adults with OCD with combined MCT and a medication (fluvoxamine) condition has also provided evidence supporting the intervention (Shareh et al., 2010). These studies obtained clinically significant results equal or better to those typically found in RCTs of CBT for OCD (Fisher and Wells, 2005b). In the present evaluation, we aimed to benchmark our usual group CBT for OCD and carry out a further benchmarking analysis for group MCT to determine if this could provide any clinical advantages in a subsequent cohort of adults who attended the same service for OCD in a mental health hospital over a subsequent 5-year period.

## Materials and Methods

### Design

This is a benchmarking analysis or clinical/quality audit of a prospectively, routinely delivered clinical service involving treatment as usual (CBT) or MCT for patients with OCD. In view of this, review and approval by a relevant research ethics committee was not required according to institutional or national guidelines.

### Patients

Patients were individuals who were consecutively referred by General Practitioners or Consultant Psychiatrists to a clinical service for OCD in an independent mental health hospital in the North West of England. The suitability to attend this service offering group psychological treatment for OCD was based primarily on patients being 18 years or older and meeting primary DSM-IV (American Psychiatric Association, 1994) criteria for OCD without concurrent diagnoses of organic mental disorders, substance-related disorders, anorexia, mania or psychosis. Unlike research treatment studies, suitability for this routine service was not based on factors such as severity, comorbidity, specific treatment history, motivation, or concomitant pharmacotherapy. During the first 5-year period, a total of 181 patients were referred to the service and 172 of them agreed to attend an initial assessment of suitability. The reasons given for not attending the initial assessment were due to work/university, family, funding or unknown issues. Of the 172 patients who attended for an initial assessment, 166 patients were suitable for the service and agreed to take part in the group treatment. Of these 166 patients, 18 did not attend any of the treatment sessions due to work/university (*n* = 10), family/health (*n* = 4), funding (*n* = 2), or unknown (*n* = 2) reasons and 23 did not consent for their clinical data to be used for purposes of clinical/quality audit. Note that only the data from patients who had provided written informed consent was used for these purposes. Therefore, the group CBT cohort described here refers to the data from the 125 patients who consented and participated in the service offering group CBT for OCD over this time period. Table 1 shows the demographic and clinical characteristics of the CBT cohort.

**Table 1:** Demographic and clinical characteristics of group treatment cohorts.

	Group CBT cohort (*n* = 125)	Group MCT cohort (*n* = 95)	Test statistic
**Mean age (*SD*)**	34.98 (9.86) (range: 18–62)	31.76 (9.91) (range: 18–56)	*t*(218) = 2.39, *p* = 0.018
**Gender**			*χ*^2^(1) = 0.72, *p* = 0.396
Men	59 (47.2%)	50 (52.6%)	
Women	66 (52.8%)	45 (47.4%)	
**Referral source**			*χ*^2^(1) = 0.31, *p* = 0.579
General practitioner	10 (8%)	6 (6.3%)	
Consultant psychiatrist	115 (92%)	89 (93.7%)	
**Mean duration of OCD in years (*SD*)**	15.26 (11.29) (range: 1–46)	15.21 (10.22) (range: 1–44)	*t*(218) = 0.03, *p* = 0.975
**Mean number of Axis I disorders (*SD*)**	2.29 (0.88)	2.41 (0.78)	*t*(218) = -1.08, *p* = 0.283
**Number of comorbid disorders**			
No comorbid disorders	30 (24%)	10 (10.5%)	
One comorbid disorder	56 (44.8%)	44 (46.3%)	
Two comorbid disorders	29 (23.2%)	36 (37.9%)	
Three comorbid disorders	9 (7.2%)	4 (4.2%)	
Four comorbid disorders	1 (0.8%)	1 (1.1%)	
**Classification of comorbid disorders**			
**Anxiety disorders**	56 (44.8%)	55 (57.9%)	
Specific phobia	4 (3.2%)	1 (1.1%)	
Social phobia (social anxiety disorder)	7 (5.6%)	3 (3.2%)	
Panic disorder with/without agoraphobia	5 (4%)	4 (4.2%)	
Post-traumatic stress disorder	4 (3.2%)	2 (2.1%)	
Generalized anxiety disorder	36 (28.8%)	45 (47.4%)	
**Mood disorders**	87 (69.6%)	71 (74.7%)	
Bipolar disorder	2 (1.6%)	4 (4.2%)	
Dysthymic disorder	3 (2.4%)	2 (2.1%)	
Major depressive disorder (single episode)	55 (44%)	45 (47.4%)	
Major depressive disorder (recurrent episode)	17 (13.6%)	15 (15.8%)	
Major depressive disorder (chronic episode)	10 (8%)	5 (5.3%)	
**Bulimia nervosa**	5 (4%)	3 (3.2%)	
**Asperger’s disorder**	0	7 (7.4%)	
**Medication status at pre-treatment**			
No medication	27 (21.6%)	22 (23.2%)	*χ*^2^(1) = 0.03, *p* = 0.867
**Benzodiazepine medication**	5 (4%)	3 (3.2%)	
Diazepam (range: 2–10 mg)	5 (4%)	3 (3.2%)	
**Tricyclic Antidepressants**	8 (6.4%)	5 (5.3%)	
Clomipramine (range: 50–225 mg)	8 (6.4%)	5 (5.3%)	
**Selective serotonin reuptake inhibitors**	80 (64%)	67 (70.5%)	*χ*^2^(3) = 6.81, *p* = 0.078
Citalopram (range: 10–60 mg)	13 (10.4%)	8 (8.4%)	
Escitalopram (range: 10–20 mg)	11 (8.8%)	2 (2.1%)	
Fluoxetine (range: 20–60 mg)	11 (8.8%)	10 (10.5%)	
Sertraline (range: 25–200 mg)	45 (36%)	47 (49.5%)	
**Serotonin-norepinephrine reuptake inhibitors**	10 (8%)	4 (4.2%)	Fisher exact, *p* = 1.000
Duloxetine (range: 20–60 mg)	4 (3.2%)	2 (2.1%)	
Venlafaxine (range: 75–375 mg)	6 (4.8%)	2 (2.1%)	
**Antipsychotic medication**	19 (15.2%)	12 (12.6%)	Fisher exact, *p* = 0.146
Olanzapine (range: 5–10 mg)	2 (1.6%)	4 (4.2%)	
Quetiapine (range: 25–250 mg)	12 (9.6%)	8 (8.4%)	
Risperidone (range: 1–4 mg)	5 (4%)	0	

During the subsequent 5 years allotted to MCT, a total of 152 patients were referred to the service and 146 of them agreed to attend the initial assessment of suitability. The reasons given for not attending this initial assessment were due to work/university, illness, funding or unknown issues. Of the 146 patients who attended the initial assessment, 142 patients were suitable and agreed to take part in the group intervention. Of these 142 patients, 14 did not attend any of the sessions due to work/university (*n* = 9), family/health (*n* = 1), funding (*n* = 3), or unknown (*n* = 1) reasons and 33 patients opted out to their clinical data being used for clinical/quality audit. Note also that only the data from patients who had provided written informed consent was used for this evaluation. Therefore, the group MCT cohort described here represents the data from the 95 patients who consented and participated in the service offering group MCT for OCD over this subsequent time period. Table 1 summarizes the demographic and clinical characteristics of the MCT cohort.

### Measures

A number of self-report routine outcome measures were administered before and after each intervention. The naturalistic clinical service setting precluded collection of sufficiently appropriate long-term follow-up data, which is very common in routine clinical practice. The primary outcome measure was the severity of symptoms of OCD and the secondary outcome measures assessed depression, functional impairment, global improvement, and likelihood to recommend treatment.

### Primary Outcome Measure

The primary outcome measure was the severity of symptoms of OCD as assessed by the self-report version of the Yale-Brown Obsessive Compulsive Scale (Y-BOCS; Baer et al., 1993). The Y-BOCS is widely considered to be the “gold standard” assessment measure in treatment outcome research in OCD (Frost et al., 1995; Fisher and Wells, 2005b). It is a 10-item measure that assesses the severity of both obsessions and compulsions across five dimensions: frequency, interference, distress, resistance, and control. The Y-BOCS has good test-retest reliability and internal consistency with Cronbach alphas of 0.89 in a non-clinical sample and 0.78 in an OCD sample (Steketee et al., 1996).

### Secondary Outcome Measures

*The Beck Depression Inventory* (BDI; Beck et al., 1961) was used to assess symptoms of depression. The BDI is a widely used 21-item scale that assesses the presence and severity of depressive symptoms over the previous week using a 4-point severity scale. The reported Cronbach alpha is 0.89 (Beck et al., 1961).

*The Work and Social Adjustment Scale* (WSAS; Mundt et al., 2002) was used as a measure of functional impairment associated with OCD. The WSAS is a 5-item scale that assesses the degree of impairment in functioning over the previous week using a 9-point rating scale. The reported Cronbach alphas ranged from 0.77 to 0.90 (Pedersen et al., 2017).

In addition to the above measures, patients were asked to complete two further ratings at post-treatment. One of these ratings was a self-report adaptation of Guy (1976) clinician-rated Clinical Global Impression-Improvement (CGI-I) scale, which was developed to be used in pharmacotherapy research trials to provide brief assessments of patient improvements. In the adapted version of this scale, the Self-Ratings of Global Improvement Scale (SRGIS), asked patients to rate on a 7-point scale their response to the following: “Compared to your initial OCD problems just before you started the OCD Treatment Program, please circle a number below to indicate how much you have improved.” Patients indicated their response by choosing one of the following: 1 = *very much improved*, 2 = *much improved*, 3 = *minimally improved*, 4 = *no change*, 5 = *minimally worse*, 6 = *much worse*, 7 = *very much worse*. In the other post-treatment rating, patients were asked to “indicate how likely you are to recommend the OCD Treatment Program you have completed for someone who might be suffering from OCD” by using a rating scale ranging from 0 (*I would not recommend it*) to 100 (*I would definitely recommend it*).

### Procedure

Following referral to the clinical service for OCD, all patients were sent a pack containing the following: (1) a letter offering them “an appointment to attend an initial psychological assessment interview with a view to participating in the OCD Treatment Program” and requesting completion of enclosed measures; (2) registration and consent forms; and (3) the battery of pre-treatment measures. The consent form asked patients to decide whether or not to give the hospital permission for their “clinical data to be used anonymously for purposes of clinical/quality audit.” All patients attending this interview were assessed by the first author for suitability for the service, which involved diagnostic screening using the Structured Clinical Interview for DSM-IV Axis I Disorders - Patient Edition (SCID-I/P; First et al., 1997). If patients were suitable to attend the service, they were informed at the end of the interview and provided with details of the nature of the respective treatment, including duration, facilitation, and format. This allowed for opportunities to address any specific concerns raised by patients about treatment including apprehension about the group format or expectations about attendance and participation. The patients who agreed to take part in the group treatment were then informed about start dates and encouraged to actively focus on this treatment whilst participating. Patients who had been, or were going to be, prescribed any psychotropic medication were also encouraged to ensure that adequate clinical management of their medication from their General Practitioner and/or Consultant Psychiatrist was regularly in place throughout their group treatment participation. Patients then waited between approximately 1 day and 3 weeks before commencing treatment.

CBT and MCT were delivered in group formats jointly by the first and second authors and each group treatment consisted of 12 2-h weekly sessions over a period of 4 months. The first author is a Clinical Lead and Consultant Clinical Psychologist with extensive training and experience in CBT and MCT for OCD. The second author used to be a service-user when she initially attended for individual treatment for OCD. Since achieving full recovery following CBT 14 years ago, she has been co-facilitating each group treatment session over the entire period of the clinical service for OCD and gaining considerable experience under supervision. CBT for OCD followed the treatment approach advocated by Salkovskis and Kirk (1989, 1997) but also that of Wilhelm and Steketee (2006) in order to comprehensively extend the focus beyond inflated responsibility and to other cognitive domains implicated in OCD such as overestimation of threat, intolerance of uncertainty, and perfectionism. MCT for OCD followed the treatment approach of Wells (1997, 2009) and the published treatment protocol (Wells, 2009). The delivery rather than the content of each treatment modality was adapted for use in the group format. Common to both interventions was the content of sessions 1, 8, and 12 where the primary focus was on psychoeducation about OCD and its treatment and motivational enhancement (session 1), how significant others (a family member, friend, or colleague of each patient attended this session) could support the patient in maximizing therapeutic gains (session 8), and therapy blueprint and relapse prevention (session 12). There were other common general features of the two treatments including conceptualization, socialization, exposure to feared stimuli, and verbal and behavioral reattribution strategies were used to change beliefs and behaviors, but for each of these features the content and focus was different. Specifically, during CBT the focus was on extensively challenging relevant cognitive belief domains and implementing self-directed ERP whilst the focus during MCT was to challenge metacognitive beliefs in OCD (i.e., metacognitive beliefs about intrusions and beliefs about rituals and stop signals). In addition, during MCT patients were introduced to detached mindfulness as an alternative means of responding to their intrusions and instructed to postpone worry and rumination. MCT implemented metacognitively focused exposure aimed at testing fusion beliefs. At session 12, all patients were re-administered the Y-BOCS, BDI, and WSAS and they were also asked to provide ratings of global improvement using the SRGIS and ratings of likelihood to recommend treatment.

### Overview of Analyses

We examined the outcomes of the group CBT and the group MCT against other previous research treatment studies of CBT to gauge the relative effects of these interventions when delivered in routine clinical practice. Statistical analyses were conducted using within-subjects *t*-tests to examine changes in outcome variables within each group treatment. Mixed model ANCOVAs were computed to examine differences in improvement in Y-BOCS between the CBT and MCT interventions. These were followed by between-group ANCOVAs on post-treatment variables. In non-randomized evaluations like this, it is important to control for potential threats to internal validity that are not minimized by a randomization method. Therefore, we controlled for the following pre-treatment factors: age, gender, number of diagnoses, symptoms of depression, and medication status in all of the mixed model analyses with additional controls of the pre-treatment Y-BOCS in the post-treatment between-groups ANCOVAs. We did not control for WSAS when assessing Y-BOCS outcomes because of the measurement overlap as both scales assess interference or disability associated with OCD. Of most relevance to service provision, the clinical significance of the effects of each treatment was examined and compared using international expert consensus criteria for OCD.

## Results

### Benchmarking of Treatment Outcomes for the Group CBT Cohort

The demographic and clinical characteristics of the CBT cohort are shown in Table 1. In comparison to those reported in previous RCTs and other research treatment studies of CBT for OCD (for reviews, see Jónsson and Hougaard, 2009; Öst et al., 2015), the group CBT cohort had a more balanced gender distribution, considerably higher number of referrals from secondary care (i.e., Consultant Psychiatrists), more comorbidity, and greater number of patients who were prescribed psychotropic medication. The remaining demographic and clinical characteristics of the CBT cohort were consistent with previously published data. On the whole, our CBT cohort seemed to be a group of patients with more complex OCD presentations than those previously reported in treatment studies.

We next examined the attrition rates for the entire course of CBT. Attrition was defined as a patient who takes part in at least the first group treatment session, but then withdraws before completion of the intervention (Öst et al., 2015). During the 5-year course of the group CBT, 12 (9.6%) patients dropped out of this intervention. This compares relatively well to the previously reported drop out rates, which have ranged from 11.4% for CT to 32% for the combined ERP, CT and antidepressant medication (Öst et al., 2015). The analyses presented here are based on intention to treat. Therefore, the attrition rate for the group CBT suggests that patients found this intervention acceptable. In addition, the mean number of group CBT sessions attended was 11.42 (*SD* = 0.86, range: 8–12) in mean group sizes of 7.7 (*SD* = 1.71, range: 6–11), and both of these sets of data are consistent with those reported in previous group CBT for OCD studies (Jónsson and Hougaard, 2009).

The descriptive and summary statistics for the primary and secondary outcome measures before and after each group CBT intervention are presented in Table 2. At pre-treatment, the CBT cohort displayed mean Y-BOCS scores indicating severe obsessive-compulsive symptoms and the mean BDI and WSAS scores were suggestive of moderate levels of depression and functional impairments, respectively. As shown in Table 2, all of these scores decreased from pre-treatment to post-treatment. The repeated measures *t*-tests indicated that these within-group changes were all significant in terms of Y-BOCS [*t*(124) = 24.52, *p* < 0.0005], BDI [*t*(124) = 13.35, *p* < 0.0005], and WSAS [*t*(124) = 12.35, *p* < 0.0005].

**Table 2:** Means, *SD* (in parentheses), and summary statistics for the primary and secondary outcome measures before and after each group treatment.

Measure	Group CBT cohort (*n* = 125)	Group MCT cohort (*n* = 95)	Test statistic
**Y-BOCS**			
Pre-treatment	24.94 (4.98)	26.71 (5.07)	*t*(218) = -2.58, *p* = 0.011
Post-treatment	11.66 (6.11)	11.48 (5.43)	*t*(218) = 0.22, *p* = 0.829
**BDI**			
Pre-treatment	19.78 (10.66)	22.38 (10.22)	*t*(218) = -1.83, *p* = 0.035
Post-treatment	9.11 (8.64)	10.57 (9.69)	*t*(218) = -1.17, *p* = 0.121
**WSAS**			
Pre-treatment	20.74 (9.2)	24.51 (8.4)	*t*(218) = -3.12, *p* = 0.001
Post-treatment	10.65 (9.03)	11.55 (8.81)	*t*(218) = -0.74, *p* = 0.231

*Y-BOCS, self-report version of the Yale-Brown Obsessive Compulsive Scale; BDI, Beck Depression Inventory; WSAS, Work and Social Adjustment Scale*.

It is well-known that antidepressant medication is effective in the treatment of OCD (e.g., Soomro et al., 2008). Therefore, because a large proportion of the patients in the CBT cohort were taking medication, we examined within-group pre-post effect sizes (Hedges’ *g*) based on the Y-BOCS scores for those with and without medication to estimate any effects associated with the combined treatment. In the medicated sub-group (*n* = 98), the effect size was 2.39 compared to the non-medicated sub-group (*n* = 27), which was 2.59. It is important to note that the medicated sub-group displayed more severe pre-treatment symptoms of OCD that the non-medicated sub-group.

For the entire CBT cohort, the resulting pre-post Y-BOCS change score was 13.28. This compares favorably to those reported in previous CBT for OCD studies, which have ranged from 5 to 12.6 (Jónsson and Hougaard, 2009). In addition, the within-group pre-post effect size (Hedges’ *g*) based on the Y-BOCS scores was very large (ES = 2.38), and this was also comparable to those previously reported, which have ranged from 1.47 for medication, 2.06 for ERP, 2.21 for CT, and 2.95 for combined ERP, CT and medication (Öst et al., 2015). We computed the self-ratings of global improvement data and the results indicated that 25 (20%) of patients rated their improvement following group CBT as “very much improved,” 65 (52%) as “much improved,” and 35 (28%) as “minimally improved.”

Finally, we examined the extent (0–100%) to which patients were likely to recommend the group CBT for someone who might be suffering from OCD. At post-treatment, the mean score for this scale was 92.96 (*SD* = 11.71). This indicates a significant degree of satisfaction with the treatment experienced, as patients were highly likely to recommend it.

### Benchmarking of Treatment Outcomes for the Group MCT Cohort

Table 1 displays the demographic and clinical characteristics of the group MCT cohort. In comparison to those reported in previous studies of CBT for OCD (Jónsson and Hougaard, 2009; Öst et al., 2015), the MCT cohort was slightly younger, had a more balanced gender distribution, substantially more referrals from secondary care, higher comorbidity, and more patients taking psychotropic medication. The duration of OCD was consistent with previously reported data. Table 1 shows that compared to the CBT cohort, the MCT cohort was significantly younger. Therefore, the group MCT cohort also seemed to be a group of patients with more complex OCD presentations than those previously reported in treatment studies.

During the 5-years running of group MCT, 7 patients (7.4%) dropped out of this therapy. This compares relatively well to the previously reported drop out rates in CT and for the combined ERP, CT, and medication treatment, but also to the drop out rates found for our CBT cohort although there was no significant difference in drop out rates between the treatment cohorts [*χ*^2^(1) = 0.58, *p* = 0.447]. The analyses shown here are based on intention to treat. Therefore, this attrition rate indicates that patients found this intervention acceptable. In addition, the mean number of group MCT sessions attended was 11.33 (*SD* = 0.95, range: 8–12) with mean group sizes of 7.75 (*SD* = 1.85, range: 5–10), and both of these sets of data were comparable with those published in previous group CBT for OCD studies (Jónsson and Hougaard, 2009).

Table 2 shows that at pre-treatment the MCT cohort displayed mean Y-BOCS scores indicating severe obsessive-compulsive symptoms and the mean BDI and WSAS scores were suggestive of moderate levels of depression and functional impairments, respectively. As shown in Table 2, all of these scores decreased from pre-treatment to post-treatment. The repeated measures *t*-tests indicated that these within-group changes were all significant in terms of Y-BOCS [*t*(94) = 24.06, *p* < 0.0005], BDI [*t*(94) = 11.52, *p* < 0.0005], and WSAS [*t*(94) = 13.66, *p* < 0.0005].

Similar to the CBT cohort, a large proportion of the patients in the MCT cohort were taking medication. Therefore, we also examined within-group pre-post effect sizes (Hedges’ *g*) based on the Y-BOCS scores for those with and without medication to estimate any effects associated with the combined treatment. In the medicated sub-group (*n* = 75), the effect size was 2.81 compared to the non-medicated sub-group (*n* = 20), which was 3.57. It is also noteworthy that the medicated sub-group displayed more severe pre-treatment symptoms of OCD that the non-medicated sub-group.

For the entire MCT cohort, the pre-post Y-BOCS change score was 15.23, which is highly comparable to those reported in previous CBT for OCD studies (Jónsson and Hougaard, 2009). In addition, the within-group MCT pre-post effect size (Hedges’ *g*) based on the Y-BOCS scores was very large (ES = 2.89), which was comparable to those previously reported, almost equating to the ES of 2.95 for the combined ERP, CT and medication (Öst et al., 2015). We then computed the SRGIS data and the analyses indicated that 24 (25.3%) of patients rated their improvement after the group MCT as “very much improved,” 62 (65.3%) as “much improved,” and 9 (9.4%) as “minimally improved.”

Finally, we examined the extent to which patients were likely to recommend the group MCT for someone who might be suffering from OCD. At post-treatment, the mean score for this scale was 95.26 (*SD* = 8.1). This indicates a significant degree of satisfaction with the treatment experienced, as patients were highly likely to recommend it.

### Comparisons Between Group CBT and Group MCT on Primary and Secondary Variables

There were no significant differences between the CBT and MCT cohorts in terms of mean number of sessions attended [*t*(218) = 0.79, *p* = 0.214] or the mean group sizes [*t*(218) = -0.21, *p* = 0.416]. When comparing patients’ ratings of likelihood to recommend treatment, the analyses indicated that there was no significant difference between the group CBT and group MCT in terms of these ratings [*t*(218) = -1.64, *p* = 0.062)]. This would imply that any actual outcome differences between the two group treatments are less likely to be due to non-specific factors such as satisfaction, acceptability or credibility. However, as Tables 1, 2 show there were significant pre-treatment differences in terms of age, Y-BOCS, BDI, and WSAS.

A mixed model ANCOVA with cohort (CBT vs. MCT) as the between-groups factor and time (pre-treatment and post-treatment) as the repeated-measures factor was computed on the primary outcome variable (i.e., Y-BOCS). The covariates were the following pre-treatment variables: age, gender, number of diagnoses, BDI, and medication status. There was a significant interaction involving group and time [*F*(1, 213) = 4.03, *p* = 0.046], which showed that the MCT cohort improved significantly more than the CBT cohort over the 12-week course of treatment. Follow-up between-group ANCOVA controlling for pre-treatment Y-BOCS and all other covariates (i.e., age, gender, number of diagnoses, BDI, and medication) showed no significant post-treatment group differences on Y-BOCS score [*F*(1, 212) = 1.09, *p* = 0.296]. Therefore, at post-treatment the Y-BOCS scores were similar but the MCT cohort showed a greater level of improvement than the CBT cohort over time.

When statistically controlling for pre-treatment age, gender, number of diagnoses, BDI, and medication status, mixed model ANCOVAs demonstrated no significant group by time effects on the following post-treatment outcomes: BDI [*F*(1, 214) = 0.79, *p* = 0.374] and WSAS [*F*(1, 213) = 3.69, *p* = 0.056]. Moreover, when controlling for WSAS at pre-treatment and all other pre-treatment variables, follow-up ANCOVAs on post-treatment WSAS score showed that the group effect was not significant [*F*(1, 212) = 0.47, *p* = 0.494]. Similarly, for the BDI at post-treatment when controlling for pre-treatment BDI, Y-BOCS and the other covariates, the group effect was not significant [*F*(1, 212) = 0.04, *p* = 0.852]. However, when statistically controlling for pre-treatment Y-BOCS, age, gender, number of diagnoses, BDI, and medication, ANCOVA on the patients’ ratings of global improvement indicated greater improvement following MCT than CBT [*F*(1, 212) = 8.37, *p* = 0.004].

In order to examine the relative clinical significance of the group interventions, we applied the international expert consensus criteria for defining treatment response, remission, and recovery in OCD (Mataix-Cols et al., 2016). The consensus definitions involve a twofold criterion and can be operationalized as follows: response is defined as a ≥35% reduction in Y-BOCS scores plus CGI-I rating of 1 (“very much improved”) or 2 (“much improved”) lasting for at least 1 week; partial response is defined as a ≥25% but <35% reduction in Y-BOCS scores plus CGI-I rating of at least 3 (“minimally improved”) lasting for at least 1 week; and it is assumed that no response is defined as <25% reduction in Y-BOCS scores. In the absence of CGI-I ratings, we relied on the patients’ ratings of global improvement using the SRGIS, which maintains the same criteria. However, we were unable to apply the criteria to estimate rates of remission, recovery, or relapse as the criteria required the Clinical Global Impression-Severity (CGI-S) ratings, 1- and 12-month follow-up data, which we did not have available. Table 3 displays the proportion of patients achieving criteria for response on Y-BOCS at post-treatment for the group treatment cohorts. The MCT cohort displayed an overall higher clinical response rate than the CBT cohort and this difference was significant [*χ*^2^(2) = 12.97, *p* = 0.0015].

**Table 3:** Proportion of patients achieving criteria for response, partial response and no response on Y-BOCS scores at post-treatment for the group treatment cohorts.

	Group CBT cohort (*n* = 125)	Group MCT cohort (*n* = 95)
No response	21 (16.8%)	6 (6.3%)
Partial response	24 (19.2%)	7 (7.4%)
Response	80 (64%)	82 (86.3%)

*Based on international expert consensus criteria for OCD (Mataix-Cols et al., 2016)*.

### Treatment Resource Requirements

In routine clinical services, especially those with scarce resources, the amount of treatment required to achieve a clinical response or significant clinical improvement is an important economic factor. An advantage of group treatment delivery is that a higher volume of patients can be treated over a specified period of time. Therefore, using previous formulae (i.e., number of treatment sessions x number of hours per treatment session x number of therapists divided by number of patients per group) for calculating basic cost-savings (Jónsson and Hougaard, 2009), we estimated the mean number of therapist hours required to treat each patient in each group treatment cohort. For the CBT cohort, with two therapists treating groups with a mean size of 7.7, 2 h per week over 12 weeks, equates to a total 6.23 h per patient to achieve a 64% clinical responder rate. For the MCT cohort, with two therapists treating groups with a mean size of 7.75, 2 h per week over 12 weeks, equates to 6.19 h per patient to achieve an 86.3% clinical responder rate. Clearly, if only one therapist facilitates each group session over the course of treatment, then the mean number of hours needed to treat each patient becomes 3.12 and 3.10 for the group CBT and group MCT, respectively. Both group treatments could potentially create considerably greater cost-effectiveness when compared with individual therapy although it must be noted that the longer-term effects have yet to be established.

## Discussion

Given that CBT is the recommended treatment of choice for OCD, but few systematic studies have documented whether the results based on this recommendation can be translated into real-world settings, our first aim was to benchmark outcomes for group CBT in a routine clinical service. In a large group of patients with relatively more complex OCD presentations than previously reported, the results demonstrated that a 12-week course of group CBT led to significant improvements in OCD, depression, and functional impairments. At post-treatment, the scores from primary and secondary outcome measures fell within normal/mild ranges. The results of benchmarking indicated that the outcomes of group CBT were equal to those found in research treatment studies (Jónsson and Hougaard, 2009; Öst et al., 2015). Of particular relevance to the results obtained is the low attrition rate found given that 78.4% of patients in the CBT cohort were prescribed medication. Studies have reported that treatments with medication alone or in combination with ERP or CT tend to produce the highest attrition rates (Öst et al., 2015). The results of this benchmarking evaluation contribute to the generalizability of the findings from research treatment studies but extend it to group CBT and more complex OCD presentations.

The results of our initial benchmarking analysis based on patients’ ratings of global improvement revealed that 28% of the patients who had received group CBT reported only minimal improvement. This finding is not surprising, and consistent with literature showing that a significant proportion of patients have a minimal or no response to CBT for OCD (e.g., Wilhelm, 2000; Fisher and Wells, 2005b). It supported our second aim to address limitations by introducing MCT for OCD and examining its comparative effectiveness. The results demonstrated that a 12-week course of group MCT led to significant improvements in OCD, depression, and functional impairments. At post-treatment, the scores from primary and secondary outcome measures fell within normal/mild ranges. The clinically significant results obtained for the group MCT appeared to be better or equal to the group CBT cohort and those typically found in RCTs of CBT for OCD, especially given the low attrition rate in a group for whom 76.8% were prescribed medication. The results of the group MCT also contribute to a growing body of empirical evidence attesting to the effectiveness of this intervention in group formats for generalized anxiety disorder in children (Esbjørn et al., 2018) and adults (Van der Heiden et al., 2013; McEvoy et al., 2015), depression (Dammen et al., 2015), antidepressant and CBT-resistant depression (Papageorgiou and Wells, 2015), and transdiagnostic patient samples (Capobianco et al., 2018).

During the course of each intervention, patients found both group treatments equally and highly acceptable and satisfactory as evidenced by equivalent low attrition rates and lack of significant differences in the number of sessions attended and the patients’ treatment recommendations. However, the effect size for the MCT cohort was higher than that obtained for the CBT cohort and the patients’ ratings of global improvement coupled with treatment response rates suggests that patients receiving MCT benefitted more from this intervention. The results show that the MCT cohort improved significantly more over the 12-week course than the CBT cohort after controlling for important pre-treatment variables including age, gender, number of diagnoses, symptoms of depression, and medication. Therefore, even though the Y-BOCS scores of the treatment cohorts were similar at post-treatment, the MCT cohort displayed a greater level of improvement than the CBT cohort over time. This is likely to be due to the MCT cohort having higher scores at pre-treatment as control of pre-treatment Y-BOCS in the post-treatment analysis showed no differences between the conditions in final level of Y-BOCS score.

The clinical significance of the comparative findings is the most informative given the motivation to reduce the number of patients showing minimal or no response to treatment. Using the twofold international expert consensus criteria (Mataix-Cols et al., 2016) applied to the Y-BOCS and SRGIS, there was a reduction following MCT of 10.5% in non-responders when compared with CBT, a reduction of partial responders by 11.8% but an increase in clinical responders by 22.3%. The difference in response rates was statistically significant.

Our analyses represent a naturalistic evaluation to benchmark treatment outcomes but the obvious limitations of the present evaluation are associated with the strengths of RCTs and other research treatment studies. That is, apart from the SCID-I/P, there was a lack of clinician-administered tools, untreated control conditions, treatment fidelity and adherence checks, and independent raters or assessors. Importantly, we were unable to control for type of pharmacotherapy, which would have enabled us to determine the impact of different drugs on outcome. However, examination of within-group treatment effect sizes for patients without medication suggests a greater change in MCT compared to CBT, but these sub-group analyses are based on a small number of patients. Finally, we were not able to collect any meaningful follow-up data due to the routine clinical nature of the service within an independent mental health hospital. Nevertheless, the data are likely to represent the types of outcomes that can be achieved in clinical settings.

In conclusion, both CBT and MCT were effective interventions when delivered as group treatments in a naturalistic clinical setting. We found that MCT appeared to show some advantage over CBT. Most notably, when compared to CBT, MCT appeared to reduce the rate of non-responders and partial responders whilst significantly increasing the rates of clinical response.

## Ethics Statement

This study is a benchmarking analysis or clinical audit of a prospectively, routinely delivered clinical service rather than a research treatment study. All patients in this paper provided consent for their clinical data to be used anonymously for purposes of clinical/quality audit.

## Author Contributions

CP, KC, ST, HW, and JH contributed to the development of the service. CP, KC, ST, HW, JH, and LH contributed to the data collection, scoring, and recording. AW, CP, and LH contributed to the data analysis and interpretation. All authors contributed to writing the manuscript.

## Conflict of Interest Statement

The authors declare that the research was conducted in the absence of any commercial or financial relationships that could be construed as a potential conflict of interest.
